# Wing morphology variations in *Culicoides circumscriptus* from France

**DOI:** 10.3389/fvets.2023.1089772

**Published:** 2023-04-24

**Authors:** Leila Hadj-Henni, Zoubir Djerada, Christine Millot, Mireille Cousinat, Véronique Lehrter, Denis Augot

**Affiliations:** ^1^Usc Vecpar-ANSES LSA, EA 7510, SFR Cap Santé, Université de Reims Champagne-Ardenne, Reims Cedex, France; ^2^Department of Medical Pharmacology, EA 3801, SFR Cap Santé, Reims University Hospital, Reims Cedex, France; ^3^Université de Reims Champagne-Ardenne, Unité BioSpecT, EA7506, SFR Cap Santé, UFR de Pharmacie, Reims, France; ^4^ANSES, INRAe, ENVA, UMR-BIPAR, Laboratoire de Santé Animale, Maisons-Alfort Cedex, France

**Keywords:** geometric morphometrics, landmark, outline, *Culicoides*, wing, population

## Abstract

The biting midge *Culicoides circumscriptus* Kieffer, 1918 is a European widespread vector of avian malaria throughout the continent and is a possible vector of Akabane virus and Bluetongue virus. This species populates a wide range of environments in contrasting ecological settings often exposed to strong seasonal fluctuations. The main goals of this study were to investigate *C. circumscriptus* phenotypic variation at three departments in France (Corsica Island, Moselle and Var) and to determine if its phenotypes vary with the environment. *Culicoides circumscriptus* wing phenotypes were analyzed using a geometric morphometric approach based on anatomical landmarks and outlines of the wing. Dendogram trees based on landmarks and the outlines-2 set (cell m4) showed similar topologies and separated populations of *C. circumscriptus*. In contrast, another set of outlines-1 (covering the r-m cross vein, M, radiale and arculus) presented a different hierarchical clustering tree. The phenotypic variation observed in *C. circumscriptus* indicated that these populations are exposed to environmental and ecological pressures. Our results suggest the presence of phenotypic plasticity in this species.

## Highlights

- We applied a geometric morphometrics approach to *Culicoides* midge populations.
- This approach revealed phenotypic variation in *Culicoides circumscriptus.*
- Geometric morphometrics discriminates southern from northeastern French populations.
- Landmarks and outlines-covering the r-m cross vein, M, radiale and arculus; and cell m4 of wings gave similar results.

## Introduction

*Culicoides* biting midges (Diptera: Ceratopogonidae) play a central role in the transmission of pathogens—including viruses, filarial nematodes, and protozoans—to humans, livestock, and wildlife (1, 2). In the past years, *Culicoides* species have been involved in the spread of three major arboviruses around the world: Bluetongue Virus (BTV), Schmallenberg Virus (SBV) and Oropouche Virus (ORV) (2, 3). In 2018, Yavru and others (4) detected BTV for the first time in field-collected *C. circumscriptus* during an outbreak in Turkey. Recently, nucleic acid of Akabane virus and avian haemosporidian DNA have been detected in *C. circumscriptus, C. longipennis, C. schultzei* (5) and in *C. circumscriptus, C. impunctatus*, *C. kibunensis*, *C. paolae*, *C. pictipennis*, *C. punctatus* and *C. segnis* females (6, 7) respectively. Avian hemoprotozoa encompass different genera of blood parasites, including *Leucocytozoon, Haemoproteus* and *Plasmodium*. *Leucocytozoon caulleryi* and *Heamoproteus* spp. parasites are transmitted by *C. circumscriptus* (1, 8).

In Europe, *Culicoides* populates a wide range of environments in contrasting ecological settings often exposed to strong seasonal fluctuations.^1^ The distribution, abundance and seasonal occurrence of biting midges is determined by the availability of moisture-rich habitats that are essential for the development of immature stage. The muds are associated with aquatic or semiaquatic habitats. The composition of these muds comes from animals and vegetal detritus (9–11). Most of *Culicoides* species include anautogenous adult females (9), requiring a blood meal to produce eggs. The frequency of feeding varies with species and meteorological conditions (12, 13); host availability plays an important role in the feeding behavior of biting midges in general. Currently, biology and ecology of *C. circumscriptus* remain poorly known. Previous studies based on mitochondrial markers indicated several populations of *C. circumscriptus* (14, 15). Natural populations of *C. circumscriptus* show morphological variability in antennal *sensilla coeloconia* (16) and wing patterns (17). *Culicoides circumscriptus* shows plasticity in the type of habitat occupied for larval development, and can include sand dunes, sewage channels (sites poor in oxygen), damp sites (without surface water), salt marshes, shady areas and most livestock farming areas (18–21). In France, *C. circumscriptus* is considered as a low abundance species (22). *Culicoides circumscriptus* is abundant on Corsica island, but not in other French regions (23). The optimum temperature for *C. circumscriptus* adults is 14°C (24). At a local scale, the distribution of vector species of pathogens can change according to environmental parameters and, in turn influence disease distribution. The question is whether there are any differences in terms of phenotypic or genetic features between southern and northern French populations of *C. circumscriptus*. Our study focuses on three departments in France: Corsica Island, Moselle (North-East) and Var (South-East). The trapping sites in the South of France (Corsica and Var) were set up near horse farms and facultative summer diapause occurs during the hot periods of the year. In Moselle site, insects were collected in salt marshes, which undergo large variations during the year, from flooding (during the winter and the spring) to drought (during the summer). During drought, no specimens can be caught (Augot et al.*, comm. Pers.*). Under climate change, plastic responses involving diapause are often critical for population persistence, but key diapause responses under dry and hot conditions remain poorly understood. Thus, the beginning and the end of diapause may also play a role in the phenotypic differentiation observed in adults.

Wing geometric morphometrics (WGM) is a newly developed morphometric technique to investigate phenotypic variations (shapes and sizes) of organisms using the principles of geometry Dujardin, 2008 (25). WGM analyses can be conducted using landmarks, semi-landmarks or outline based methods (25, 26). The landmark-based approach used anatomical points (called “landmarks”); in general a small biological structure. These approaches compared the relative position of landmarks (size and shape) on several individuals. The outline-based is generally restricted to closed contours (called “outlines”) where anatomical landmarks are lacking. The outline-based approach evaluated the size and the shape describing contours of forms (27). Insect wings are the most appropriate structures for geometric morphometric studies (28). WGM is largely developed in several vectors families like as Culicidae [see review of Lorentz et al. (29)] to explore intraspecific variations among mosquito populations or to research interspecific variation, to study in sexual dimorphism, plasticity and deviation, to detection of parasites and to characterize laboratory strain. This technique was used to study the intra specific variations in Glossinidae (30), Muscidae (31), Psychodiae (28, 32, 33), Reduviidae (34) and Tabanidae (35, 36) and to show inter specific variations in Muscidae (37). The landmark-based WGM analysis of *Culicoides* wings has proven to be a valuable tool for interspecific discrimination (38–42), *C. circumscriptus* intersexes specimens (43), sexual dimorphism (44) and geographic variations (45).

A better knowledge of *C. circumscriptus* is advisable because this species is involved in the transmission of pathogens. Here, we investigated the morphological variation of French populations on a quantitative basis. The main aim here is to assess the intraspecific phenotypic variability of *C. circumscriptus* at a population level using landmark and outlines based on WGM methods, and to evaluate the intrapopulation wing shape and size variabilities. More specifically, we compared the efficiency of anatomical landmarks and outlines of the wing to separate populations. This research will serve as a guideline for choosing the best WGM landmark set (s) for separating populations in the field. Moreover, for comparison, a molecular approach based on the DNA mitochondrial (mtDNA) cytochrome C oxidase I (Cox1) gene was used to distinguish between populations.

## Materials and methods

**Figure 1:**
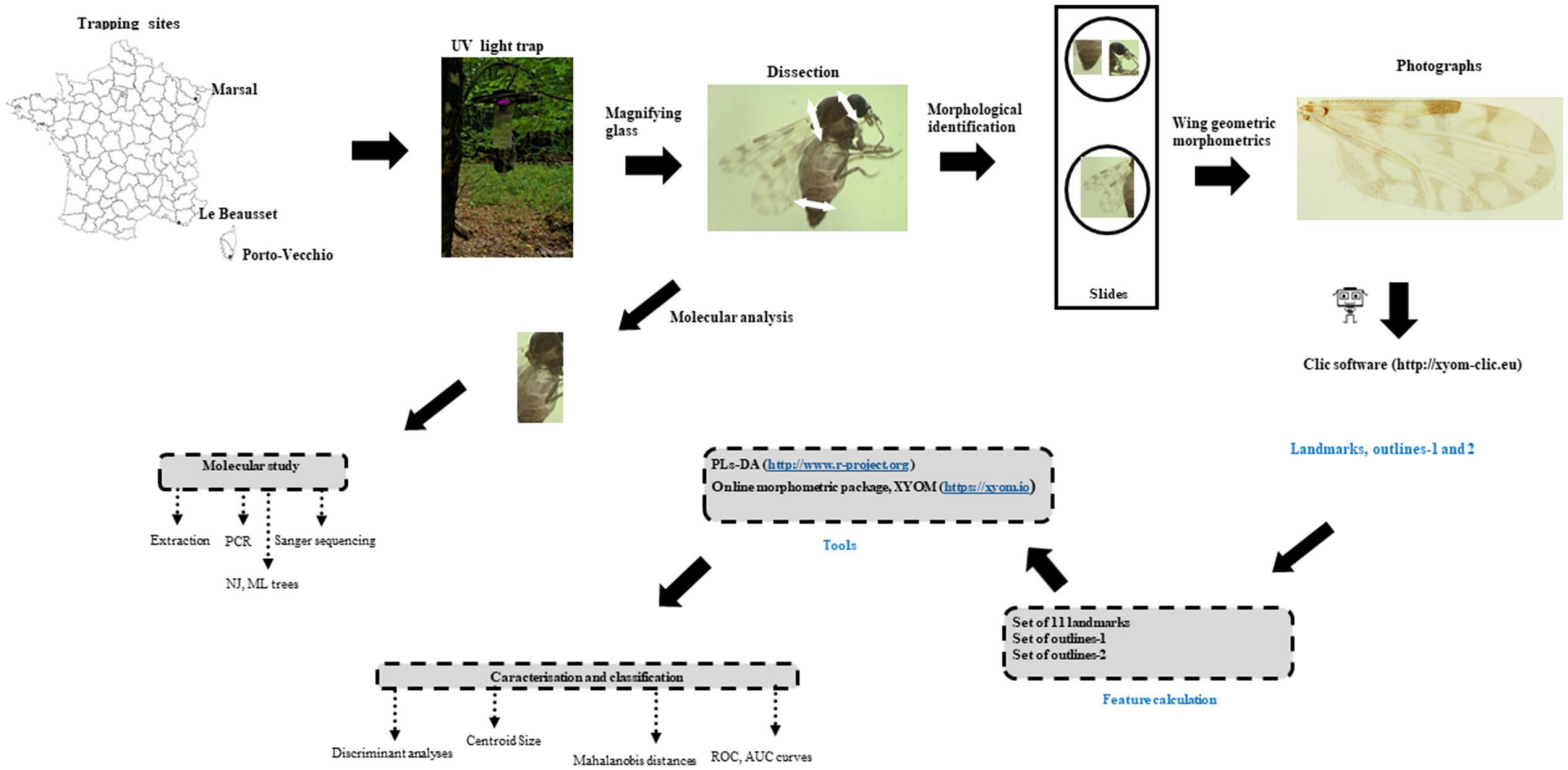
Block diagram of the study.

The workflow of the entire process is shown in Figure 1.

### Sample material

The three study sites were located in Porto-Vecchio (41°35′30″N; 9°16′49″E), Corsica (collected in July 2015), Marsal (48°47′24″N; 6°36′35″E), Moselle and Le Beausset (43°11′56″N; 5°48′12″E), Var (collected in June 2010; Figure 1; Table 1). In Var (*n* = 24) and Corsica (*n* = 20), insects were caught using UV light traps near horse farms; in Moselle (*n* = 22), specimens came from soil samples collected in a salt marsh (46) near cattle farms. Soil samples (water and underling soil) were collected haphazardly in salt marsh between March and June 2018 and 2019 according to *Culicoides* abundance (47). Soil samples placed into plastic buckets were stored in individual netted cage in the laboratory at 22°C (71.6°F). Tap water was added regularly to keep samples humid but not waterlogged (48). The adults that hatched rose toward the light and could thus be easily harvested with a mouth-operated aspirator or an Eppendorf tube. Emerging *Culicoides* were collected two or three times a week (48). Trapping and emerging adults were stored in 70% ethanol before mounted. All females were identified under the microscope according to morphological characters (49) and each individual specimen was mounted in Euparal® medium with head, wings and genitalia (50) (Figure 1).

**Table 1:** Description of the sampling stations and number of *Culicoides circumscriptus* wings analyzed by site for the geometric morphometrics analysis.

Site	Coordinates		Biotope	Number of wings analyzed by category		
	North	East		Landmarks	Outlines-1	Outlines-2
Corsica	41°35′30″	9°16′49″	Rural area, near horse farms	20	20	20
Moselle	48°47′24″	6°36′35″	Salt marsh, rural area, near cattle farms	20	21	22
Var	43°11′56″	5°48′12″	Rural area, near horse farms	23	24	23

### Acquisition and analysis of molecular data

#### DNA extraction, amplification and genotyping

Biting midge DNA was extracted from the thorax and legs (Figure 1) using the QIAmp DNA Mini kit (Qiagen, Germany) according to the manufacturer’s recommendations (50). Polymerase chain reaction (PCR) amplification of the cytochrome oxidase subunit I (Cox1) gene region was conducted with the protocol published by Hadj-Henni et al. (41) using the primers C1J1718 (5′-GGAGGATTTGGAAATTGATTAGT-3′) and C1N2191 (5′-CAGGTAAAATTAAAATATAAACTTCTGG-3′) (51). The PCR products were visualized by gel electrophoresis in 1.5% agarose gel, stained with GelGreen (Biotium). All positive amplicons were Sanger sequenced (Genewiz, GmbH, https://www.genewiz.com).

#### Phylogenetic analysis

Cleaned PCR products were sequenced by Genewiz, GmbH (www.GENEWIZ.com). Assembly of sequences were performed using the Pregap and Gap programs included in the Staden software package (52). Additionally, the Cox1 Genbank sequences of *C. circumscriptus* populations were also included in our molecular analyses (Supplementary Table 1). Alignments and phylogenetic analysis were conducted with MEGA 7 (53). Distance analysis was performed using the neighbor-joining (NJ) method (Kimura 2-parameter = K2P). Trees were constructed using the neighbor-joining (NJ) method (Kimura-2 parameter) and the maximum likelihood (ML) method (Hasegawa-Kishino-YanoTamura 3 model); 1,000 bootstrap replicates were used to test the robustness of the constructed trees. Trees were rooted using a sequence from *Culicoides nubeculosus* (KJ624102) as an outgroup (54).

### Acquisition and statistical analysis of landmark data

#### Wing preparation

**Figure 2:**
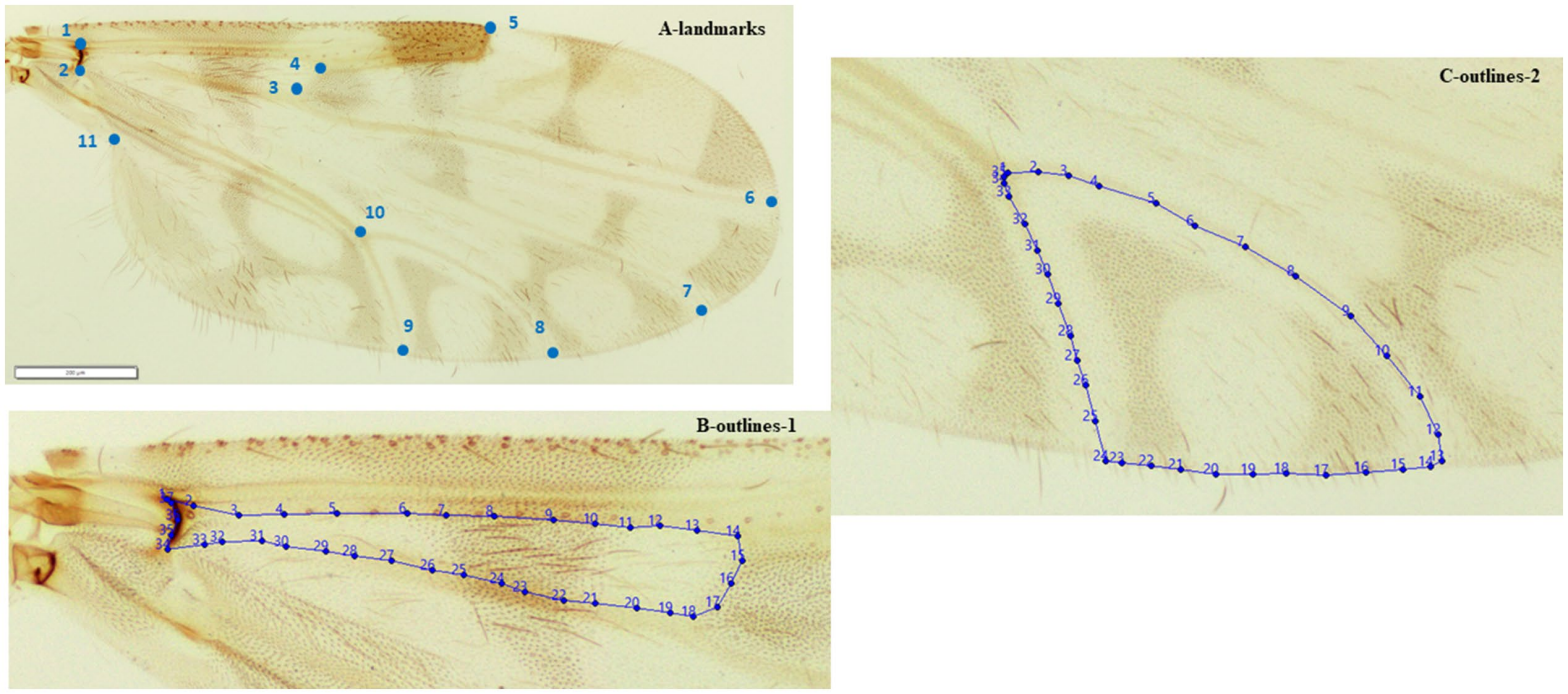
Position of the 11 landmarks **(A)** outlines-1 **(B)** outlines-2 **(C)** on the right wing of adult female *Culicoides circumscriptus* used for geometric morphometric analysis.

For the WGM analysis, the right wings from females were fixed on slides with Euparal® and flattened under cover slips (Figure 1). The differential directional asymmetric effects between left or right wing has been estimated at a 1% or 2% of the interindividual variation (55); which should not interfere with our comparisons based on one side of the biting midges. The wing samples were photographed using an Olympus BX53 microscope equipped with an Olympus SC100 camera, under 10 X magnification. A total of 66 specimens were chosen for plotting landmarks and outlines (Figure 2). A total of 11 landmarks were selected based on the ease with which they could be plotted across all *Culicoides* species (39–41). We chose two outlines sets (Figure 2). The contour of the cell between the r-m cross vein, M, radiale and arculus (defined by the landmarks 1, 2, 3, and 4) and the contour of the cell m4 (defined by the landmarks 8, 9, and 10) were selected; for terminology, see (49, 56).

#### Morphometric analysis

Anatomical landmarks and outlines were plotted and data analyses and graphical outputs were performed using both the CLIC package and the recently available online morphometric package, XYOM^2^ (57). The software aligned landmarks and outlines and then calculated the mean of the plotting. Landmarks were computed as orthogonal projections and compared together for every group. For outlines, an elliptic Fourier analysis was used to construct the shape variables, i.e., the normalized elliptic Fourier coefficients (NEF). Coordinates permit to characterize variables linked to the size and the shape separately.

For landmarks, wing size was estimated using the isometric estimator of the centroid size (CS) derived from data on coordinates (58). For outlines, three variables characterized the size: the square root area within the outlines, the perimeter, and the semi-major axis of the first ellipse. During the analyses, the centroid size was estimated by the half major axis of the first ellipse.

In both approaches, statistical comparisons of the CS among the species were performed by Oneway ANOVA and illustrated by quantile boxes. The CS difference was compared among species by a non-parametric test (1,000 runs) with Bonferroni correction at *p*-values <0.05. To test the validity of global size for accurate species identification, we used a maximum likelihood approach based validated reclassification approach (59). The allometric effect (the effect of size on shape variation) was performed by linear regression of the first (shape derived) discriminant factor on the CS, and then estimated by the determination coefficient r^2^ (36).

The visual comparison of shape changes across species was provided by the superposition of the average wing of each species. The generalized least squares Procrustes superimposition algorithm (60) was used to produce shape coordinates (partial warps), and the principal components (relative warps) (58) were used to compare samples (principal component and discriminant analyses). To assess the degree of similarity between biting midges, pairwise Mahalanobis distances between samples were calculated. To illustrate morphological divergence among populations, a hierarchical classification tree was built based on Mahalanobis distances.

#### Classification by machine learning

Principal component analysis (PCA) was used to explore the correlation between variables and machine-learning algorithms used to predict individual species based on variable values with the partial least squares discriminant analysis (PLS-DA) (41). Classical tools as ROC (receiver operating characteristic) curves, AUC (area under the curve), Kennard-Stone algorithm were used on our dataset to assess, optimize and predict the final models (41). Statistical analyses were performed using the R 3.6.0 Software (The R Foundation for Statistical Computing, http://www.r-project.org).

## Results

### Molecular analysis

Sequences obtained are available in GenBank under the following accession numbers: MW353288, MW353289, MW353291-96, MW353299-302, MW353304-08 and OQ711946-958.

**Figure 3:**
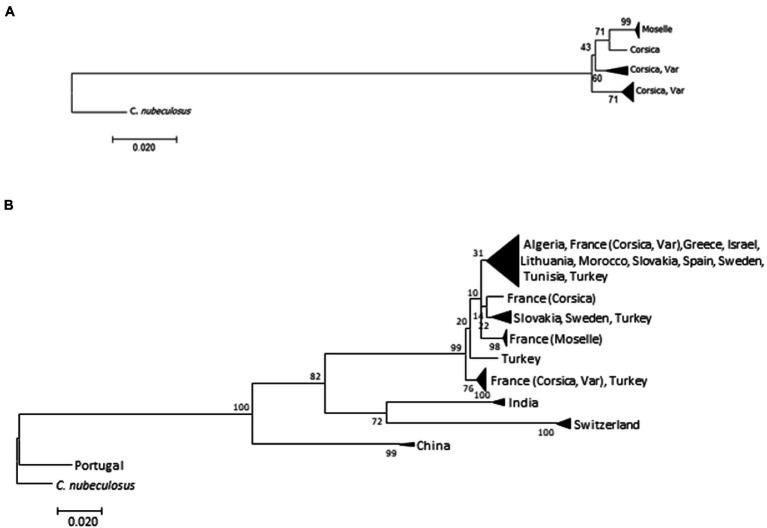
Trees obtained from the analysis of *Culicoides circumscriptus* cytochrome oxides I (COI) mitochondrial DNA using the neighbor-joining method **(A)** on our samples and the maximum-likelihood method **(B)** on both GenBank sequences and our data. Bootstrap values are shown on nodes (1,000 replicates).

ML and NJ trees were constructed, based on 401 bp, with and without *C. circumscriptus* sequences obtained from GenBank (Figure 3). The trees showed the same topology: the Moselle specimens are separated from the Corsica and Var populations (Figures 3A,B). *Culicoides circumscriptus* populations from China, India and Switzerland clustered separately from another clade with *Culicoides* specimens from North Africa and Europe (Figure 3B).

The intraspecific K2P values for the three populations is as follow: for Corsica (0.020 ± 0.005), for Moselle (0.001) and for Var (0.014 ± 0.003). The pairwise distances between our samples ranged from 0.017 (±0.004) to 0.026 (±0.007). The distances between the other populations are given in Supplementary Table 1. *Culicoides circumscriptus* from Portugal present a high intraspecific variation (>0.19). Specimens are clustered separately from other populations on the ML tree (Figure 3B).

### Classification on geometric morphometrics

#### Size variation

**Figure 4:**
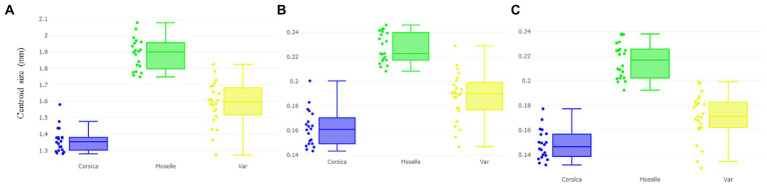
Boxplot illustrating wing size (centroid size, CS) variation based on the landmarks **(A)**, outlines-1 **(B)** outlines-2 **(C)** sets of *Culicoides circumscriptus* from three different sampling sites (Corsica, Moselle, and Var). Expand: median, percentile, and outliers.

According to CS, the largest wing was found in a female from Moselle (landmark: 1.887 mm ± 0.096; outlines-1: 0.227 mm ± 0.012; outlines-2: 0.216 mm ± 0.000), whereas the smallest wing was found in Corsica (landmark: 1.363 mm ± 0.074; outlines-1: 0.161 mm ± 0.014; outlines-2: 0.148 mm ± 0.012; Figures 4A–C).

The wing CS of *C. circumscriptus* differed significantly between all sites (Table 2) for landmarks and outlines-1 and outlines-2 (*p* < 0.05). The accuracy of the maximum likelihood validated size-based classification was very high for three landmarks types: 83% for landmarks (Corsica: 90%; Moselle: 85%; Var: 73.91%), 78% with outlines-1 (Corsica: 75%; Moselle: 90.47%; Var: 70.83%) and 82% for outlines-2 (Corsica: 75%; Moselle: 95.45%; Var: 73.91%).

**Table 2:** Non-parametric comparisons of global size estimations, 1,000 permutations (*P*-values).

Choice of landmarks		Corsica	Moselle
Landmarks	Corsica		
	Moselle	0.000*	
	Var	0.000*	0.000*
Outlines-1	Corsica		
	Moselle	0.000*	
	Var	0.011*	0.000*
Outlines-2	Corsica		
	Moselle	0.000*	
	Var	0.034*	0.000*

* Significant (*p* < 0.05).

#### Allometry

The allometric effect of *C. circumscriptus* was very important. The first and second discriminant factors (DF) derived from the Procrustes residuals were still under the influence of size (70.1% and 0.8%, respectively) after regression on centroid size with landmarks, outlines-1 (44.9% and 7.3%, respectively) and for outlines-2 (52.9% and 0% respectively).

#### Shape variation

**Figure 5:**
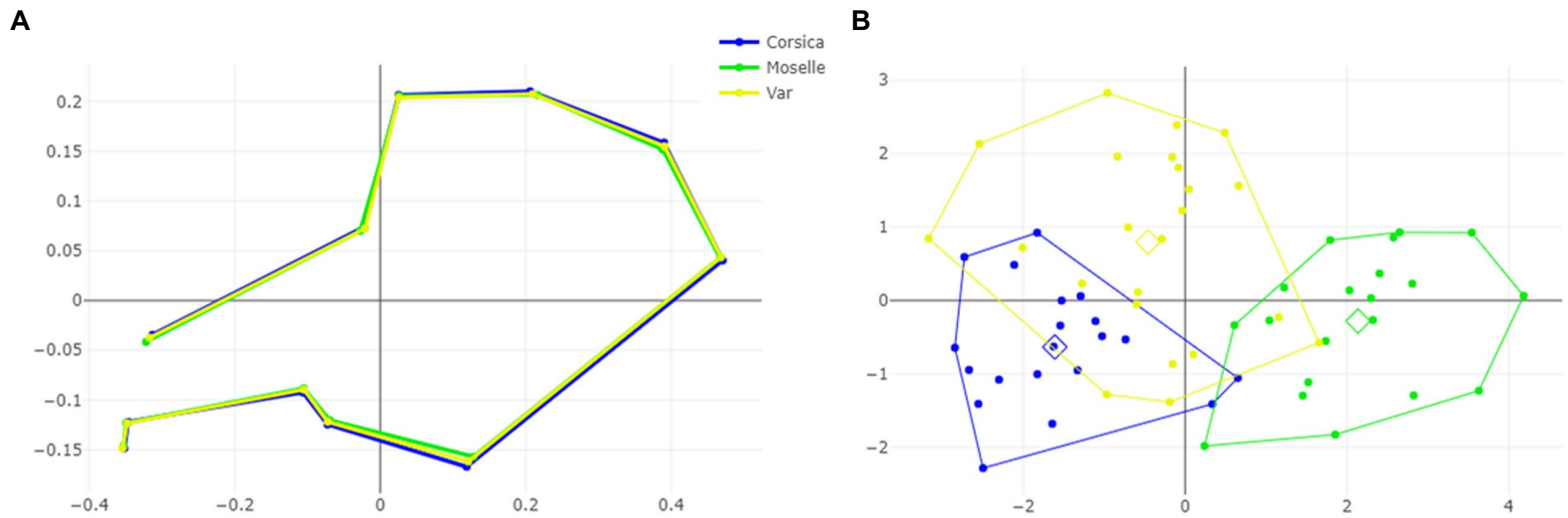
Shape variation of *Culicoides circumscriptus* based on landmarks. Superposition of the geometric morphometric set landmarks **(A)** in three geographically distant sites. Factor map of the two discriminant factors (DFs) among three sites **(B)**. Each point represents an individual. The horizontal axis is the first DF; the vertical axis is the second DF; their cumulated contributions reach 100% of the total variation.

**Figure 6:**
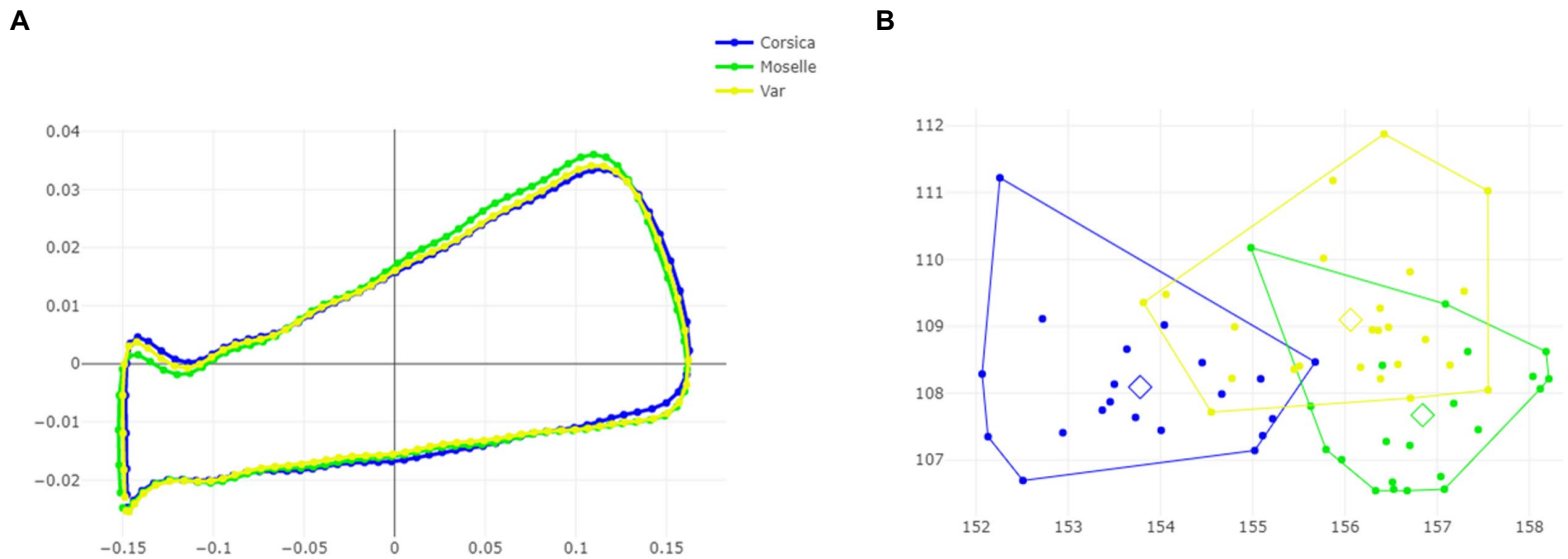
Shape variation of *Culicoides circumscriptus* based on outlines-1. Superposition of the geometric morphometric set landmarks **(A)** in three geographically distant sites. Factor map of the two discriminant factors (DFs) among three sites **(B)**. Each point represents an individual. The horizontal axis is the first DF; the vertical axis is the second DF; their cumulated contributions reach 100% of the total variation.

**Figure 7:**
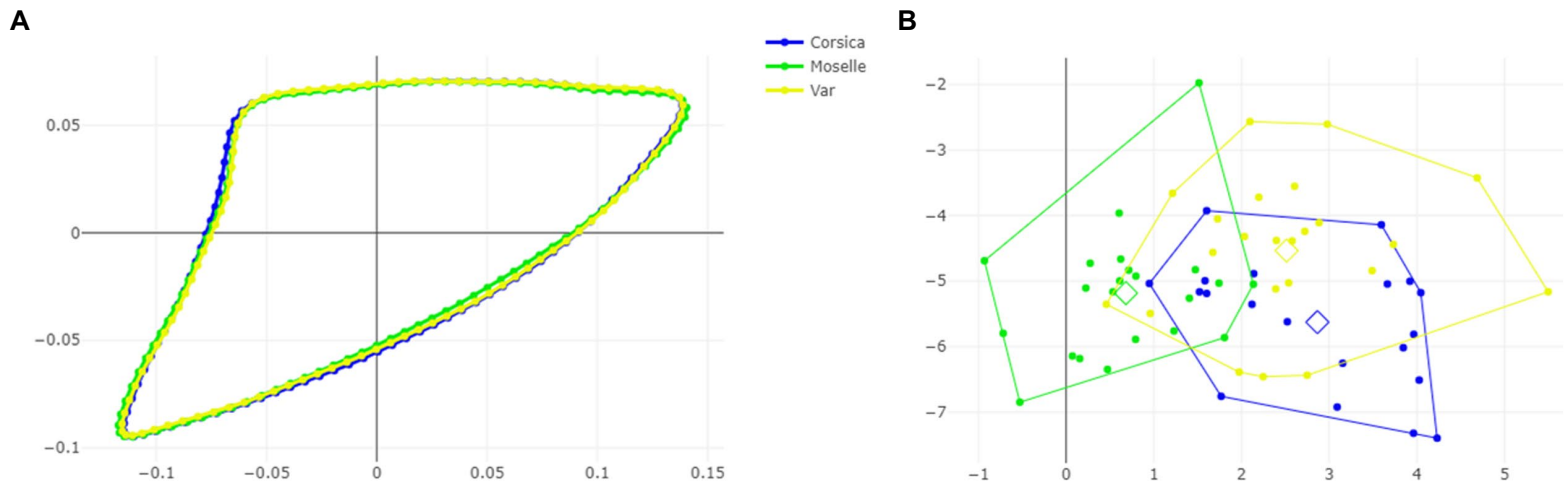
Shape variation of *Culicoides circumscriptus* based on outlines-2. Superposition of the geometric morphometric set landmarks **(A)** in three geographically distant sites. Factor map of the two discriminant factors (DFs) among three sites **(B)**. Each point represents an individual. The horizontal axis is the first DF; the vertical axis is the second DF; their cumulated contributions reach 100% of the total variation.

The visual comparisons of the mean anatomical landmark positions between populations revealed that the most visible landmark displacements were located in the upper and lower part of wing (landmarks 3, 4, 5, 7, 9, 8, 10, 11; Figure 5A). When superposing, the mean wing shape of *C. circumscriptus* between populations, the shape of Moselle appeared to be the most distinct one with outline-1 (Figure 6A) and to a lesser extent with outline-2 (Figure 7A). The discriminant analyses (DA) showed wing shape differentiation between sites and WGM approaches (Figures 5B, 6B, 7B). The DA indicated that all populations of *C. circumscriptus* were faintly overlapped (Figures 5B, 6B, 7B).

The pairwise Mahalanobis distances based on shape, landmark and outlines-1 and outlines-2, were significant (*p* < 0.05, Table 3) between Corsica and Moselle. The shape is also significant different (*p* < 0.05, Table 3) between Moselle and Var with landmarks.

**Table 3:** Mean validated reclassification scores of *Culicoides circumscriptus* populations from three French sites according to geometric morphometrics (landmarks, outlines, Mahalanobis distances for landmarks, outlines-1 and outlines-2).

Geometric morphometrics set	Sites	*n*	Classification accuracy (%)	Mahalanobis distances	
				Corsica	Moselle
Landmarks	Corsica	10/20	50		
	Moselle	17/20	85	0*	
	Var	9/23	39.13	0.117	0*
Outlines-1	Corsica	12/20	60		
	Moselle	15/21	71.42	0*	
	Var	7/24	29.16	0.003*	0.287
Outlines-2	Corsica	6/20	30		
	Moselle	15/22	68.18	0.018*	
	Var	6/23	26.08	0.867	0.064

* Significant (*p* < 0.05).

Accordingly, cross-validated classification scores of the Mahalanobis distances ranged from 26 to 85% and were highest in Moselle for landmarks (85%; Table 3). The total performance scores were 57.14%, 52.31%, and 41.54% for landmarks, outlines-1 and outlines-2, respectively.

**Figure 8:**
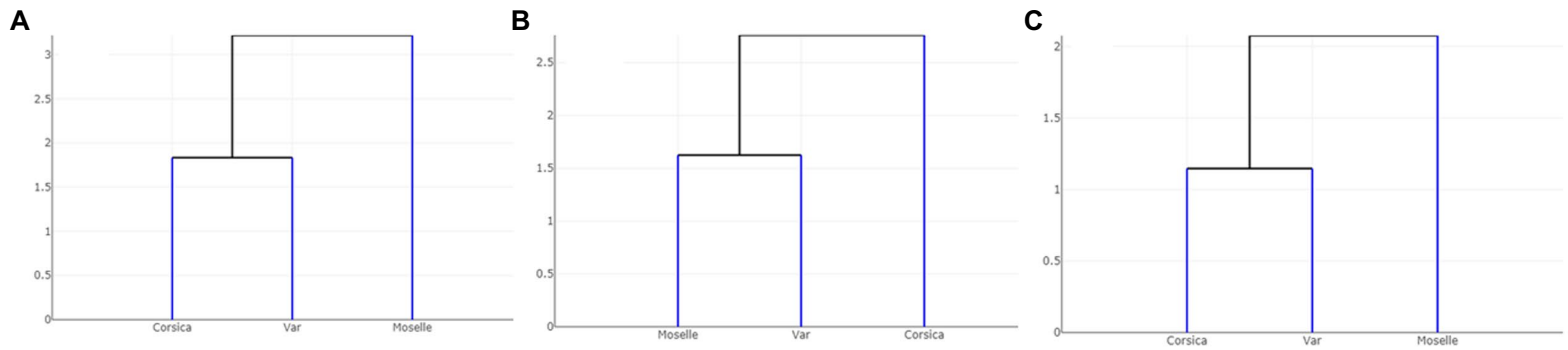
Dendogram trees based on Mahalanobis distances between populations of *Culicoides circumscriptus* computed from shape variables: **(A)** landmarks; **(B)** outlines-1 located at the apex of the wing; **(C)** outlines-2 located in cell m4 of the wing.

Dendogram trees based on Mahalanobis distances between female specimens, computed from shape variables, separated the three populations with two different topologies (Figure 8): one in which Corsica and Var populations were grouped on the same clade, separated from Moselle (landmarks and outlines-2), and one in which Var and Moselle populations were grouped together, separated from Corsica (outlines-1).

#### Classification by machine learning on geometric morphometrics

**Figure 9:**
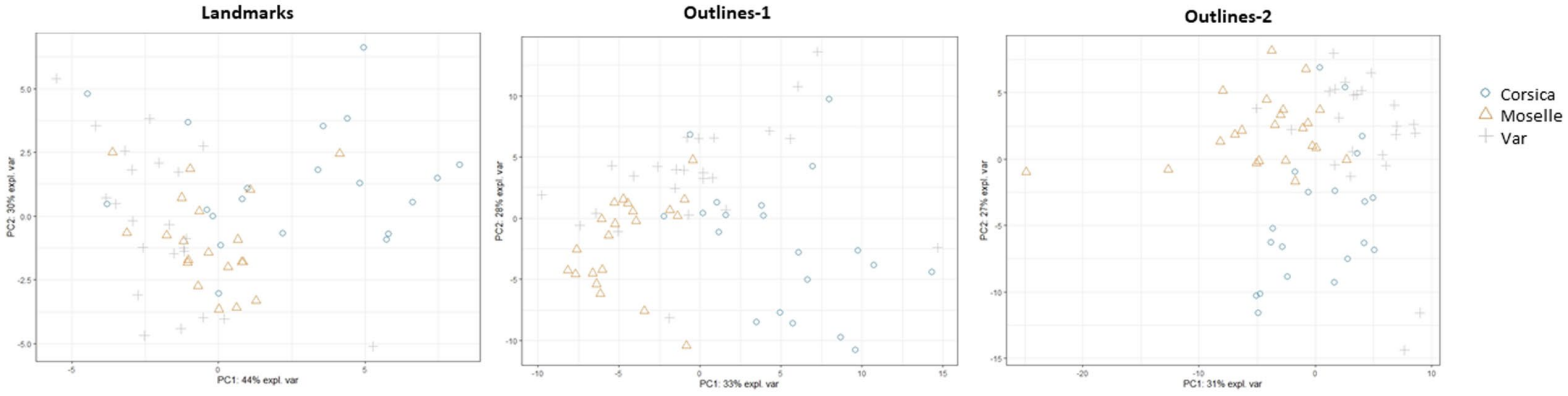
Principal component analysis (PCA) for each geometric morphometrics set.

We performed, with the PLS-DA classifier, a PCA on landmarks and outlines (Figure 9). The first two axes accounted for 44% and 30% of the variance for landmarks, 33% and 28% for outlines-1, 31% and 27% for outlines-2.

The tuning step of the number of components to select showed that 5 components were necessary to lower the balanced error rate with landmarks and outlines (-1 and-2; Supplementary Figure 1). For landmarks, the AUC values were 0.9802 (*p* = 1.073 e^−09^) for Corsica, 0.9977 (*p* = 2.621 e^−10^) for Moselle, and 0.9109 (*p* = 6.801 e^−08^) for Var. For outlines-1, the AUC values were 0.9533 (*p* = 6.668 e^−09^) for Corsica, 0.9903 (*p* = 2.091 e^−10^) for Moselle, 0.9228 (*p* = 1.561 e^−08^) for Var; for outlines-2, 0.9633 (*p* = 3.085 e^−09^) for Corsica, 0.9958 (*p* = 7.926 e^−11^) for Moselle, 0.9017 (*p* = 1.020 e^−07^) for Var. A perfect AUC of 1.0 (Table 4) was obtained for Moselle and Corsica (for outlines-1).

**Table 4:** Mean validated reclassification scores of *Culicoides circumscriptus* populations from three French sites according to classifier used (Partial Least squares discriminant—PLSDA).

Geometric morphometrics set	Sites	AUC (correctly classified rate)	*P*-value
Landmarks	Corsica	0.9965	2.883e^−10^
	Moselle	1.0000	2.164e^−10^
	Var	0.9467	4.425e^−09^
Outlines-1	Corsica	1.0000	1.595e^−10^
	Moselle	1.0000	9.127e^−11^
	Var	0.9929	4.319e^−11^
Outlines-2	Corsica	0.9989	1.750e^−10^
	Moselle	1.0000	5.474e-^11^
	Var	0.9917	7.189e^−11^

*P-value* for landmarks, outlines-1 and outlines-2.

## Discussion

This is the first WGM study on biting midges from France, here represented by three mainland and island geographic locations of *C. circumscriptus.*

By comparison of the wing based on results of the three GM methods, we found that they have the same appearance patterns for landmarks and outlines-2 (Figure 8). The outlines-1 set presented a different hierarchical clustering tree. Corresponding to the previous researches, the utilization of landmarks, semi-landmarks and outlines based on WGM show similar scores for separating species, including closely related or cryptic species (27, 61, 62). These morphometric approaches are an option to use for the species identification in studies on arthropods. The outlines-2 (cell m4) set has the advantage of being an easily recognized cell and only three landmarks delimit the contour. Moreover, the cell m4 is readily visible under a stereomicroscope and can be used directly on captured images for entomological surveillance, without mounted slides preparation. Future investigations are needed to evaluate outlines as a tool for discriminating among *Culicoides* species.

Our results show that the larger-winged population is distributed in Moselle. *Culicoides circumscriptus* exhibits changes in wing size (CS) across environments in France’s departments (Figure 4; Table 1). In insects, the wing size difference (without excluding genetic differences) is probably influenced by environmental factors (25, 63) such as temperature, relative moisture and food availability (64–66). Our results clearly show that size can be used to separate *C. circumscriptus* populations. Villard et al. (47) suggested that the *Culicoides* life-cycle depends on climatic conditions; e.g., temperature (67) but not the photoperiod (68). The larvae stages play an important part of survive of *Culicoides* during the bad climatic conditions (larval in diapause or quiescence) (68). The large wings may be favorable for finding mates, food sources and adapting to specific environments (69). *Culicoides* species are generally smaller in warm climates and larger at higher altitudes (in colder environments) according to Bergmann’s rules (70). The phenomics field (71), consists of acquiring high-dimensional phenotypic data on an organism-wide scale, and can be applied to *C. circumscriptus* using WGM.

Regarding wing shape variation, the divergence observed in Moselle site has two possible origins: the environmental effects, or the genetic drift due to geographic isolation. Moreover, the discriminant space base on shape was still affected by size variation (allometric). The influence is due mainly to the presence of a large population (Moselle, Figure 4) and it did not necessarily mean that shape variation was under the influence of environmental factors (25). Our results indicate that changes of wing shape between distant conspecific populations of *C. circumscriptus* are not a result of size variation and suggest that genetic differences may arise as species-specific adaptation to particular environments. An argument supporting that hypothesis in our sample is that, despite of significant WGM between geographic locations, there was molecular divergence between populations (Figure 3). Significantly, based on Cox1, the barcode gap between populations from Moselle and Corsica/Var is >2.5%; generally, a 2% gap is used to separate one species from the next (72). In contrast, Corsica and Var populations are grouped in different branch in our tree (Figure 3). The mtDNA is widely used for the molecular identification of species and to study their genetic diversity, including to population genetics (73). Few studies have investigated *Culicoides* sequences of Cox1 in several areas (14, 15, 74, 75). The intraspecific mtDNA genetic distances could vary considerably among species (72, 76–80) and it is difficult to give a DNA barcode distance threshold to species delimitation. Nevertheless, a high value of intraspecific distance could be indicative of the early stages of speciation. Our results show different *C. circumscriptus* populations according to morphological observations (16, 81) and molecular investigations (14, 15). Because environmental conditions in the Mediterranean Basin are comparable in terms of moisture and temperature, this similarity may account for the morphological and genetic similarity of populations captured in Var and Corsica, and the differences observed in the Moselle site.

A potential bias affecting our study could be the different collection procedures for the three populations: directly at the adult stages in Corsica and Var, after emergence in our laboratory in Moselle. Few studies have compared the phenotype between specimens collected in the field and laboratory. In mosquito, wings of wild strain of *Aedes albopictus* were significantly larger than those of the laboratory strain (82). In contrast, no information was reported between *Culex quinquefasciatus* specimens from a wild and laboratory (83). Finally, Morales et al. (65) emphasized the importance of the emerged period of *Aedes aegypti* eggs as a critical time for the size of future adults. Wild and colony fourth instar larvae (L4) of *C. sonorensis* differed in many standard metrics such as head length or width, but head ratios and pharyngeal armature measurements were comparable (84). Wild L4 appears longer than the colony. Moreover, under laboratory conditions, larval stage of *C. insignis* duration is ranged from 15.4 to 29.0 days and pupal stage ranged from 2.6 to 3.2 days (85). In our case, we have collected muds with pupae or L4 larvae stages. The emergence of the adults has been fast after the collect (<7 days) with a size bigger than biting midges collected using UV traps. Therefore, our specimens maintained during a short time in the lab, present the same parameters than wild specimens.

In conclusion, we demonstrated morphological variability in *C. circumscriptus* wing shape and size of specimens collected in southern and northeastern France. Our results support the use of WGM; landmarks and outlines-1 (covering the r-m cross vein, M, radiale and arculus) and outlines-2 (cell m4); for the morphological discrimination of populations. Differences in wing size and shape corresponded to differences in abiotic factors, and likely reflect adaptation to the environment and may furthermore affect the potential to act as vectors of disease. However, further studies on morphological differences are required to compare biting midges from different environments using standardized samples and to explore vector-borne disease transmission.

## Data availability statement

The original contributions presented in the study are included in the article/Supplementary material, further inquiries can be directed to the corresponding authors.

## Author contributions

LH-H, CM, and DA: conceptualization. LH-H, CM, MC, VL, and DA: methodology, data curation, and writing-original draft preparation. ZD and DA: software. DA: validation and resources. LH-H, CM, ZD, and DA: investigation and writing-review and editing. All authors contributed to the article and approved the submitted version.

## Funding

This study was financially supported by ANSES and Reims Champagne-Ardenne University.

## Conflict of interest

The authors declare that the research was conducted in the absence of any commercial or financial relationships that could be construed as a potential conflict of interest.

## Publisher’s note

All claims expressed in this article are solely those of the authors and do not necessarily represent those of their affiliated organizations, or those of the publisher, the editors and the reviewers. Any product that may be evaluated in this article, or claim that may be made by its manufacturer, is not guaranteed or endorsed by the publisher.

## Supplementary material

The Supplementary material for this article can be found online at: https://www.frontiersin.org/articles/10.3389/fvets.2023.1089772/full#supplementary-material

## Footnotes

^1^ www.iikculicoides.net

^2^ https://xyom.io

## References

[1] Borkent A. The biting midges, the Ceratopogonidae (Diptera) In: Marquardt WC Kondratieff BBC, editors. The biology of disease vectors. Amsterdam: Elsevier Academic Press (2005). 113–26.

[2] Carpenter S Groschup MH Garros C Felippe-Bauer ML Purse BV. *Culicoides* biting midges, arboviruses and public health in Europe. Antivir Res. (2013) 100:102–13. doi: 10.1016/j.antiviral.2013.07.020, PMID: 23933421

[3] Da Rosa JF De Souza WM De Paula PF Figueiredo ML Cardoso JF Acrani GO. Oropouche virus: clinical, epidemiological, and molecular aspects of a neglected orthobunyavirus. Am J Trop Med Hyg. (2017) 96:1019–30. doi: 10.4269/ajtmh.16-0672, PMID: 28167595 PMC5417190

[4] Yavru S Dik B Bulut O Uslu U Yapici O Kale M. New *Culicoides* vectors species for BTV transmission in central and central west of Anatolia. Annu Res Rev Biol. (2018) 27:1–9. doi: 10.9734/ARRB/2018/42170

[5] Dağalp SB Dik B Doğan F Farzani TA Ataseven VS Acar G. Akabane virus infection in eastern Mediterranean region in Turkey: *Culicoides* (Diptera: Ceratopogonidae) as a possible vector. Trop Anim Health Prod. (2021) 53:231. doi: 10.1007/s11250-021-02661-y, PMID: 33772395

[6] Veiga J Martínez-de la Puente J Václav R Figuerola J Valera F. *Culicoides paolae* and *C. circumscriptus* as potential vectors of avian haemosporidians in an arid ecosystem. Parasit Vectors. (2018) 11:524. doi: 10.1186/s13071-018-3098-8, PMID: 30269688 PMC6166282

[7] Žiegytė R Platonova E Kinderis E Mukhin A Palinauskas V Bernotienė R. *Culicoides* biting midges involved in transmission of haemoproteids. Parasit Vectors. (2021) 14:27. doi: 10.1186/s13071-020-04516-1, PMID: 33413582 PMC7789565

[8] Santiago-Alarcon D Palinauskas V Schaefer HM. Diptera vectors of avian Haemosporidian parasites: untangling parasite life cycles and their taxonomy. Biol Rev. (2012) 87:928–64. doi: 10.1111/j.1469-185X.2012.00234.x, PMID: 22616880

[9] Mellor P Boorman J Baylis M. *Culicoides* biting midges: their role as arbovirus vectors. Annu Rev Entomol. (2000) 45:307–40. doi: 10.1146/annurev.ento.45.1.307 10761580

[10] Meiswinkel R Venter GJ Nevill EM. Vectors: *Culicoides spp* In: Coetzer JAW Tustin RC, editors. Infectious diseases of livestock, vol. 1. 2nd ed. Cape Town: Oxford University Press (2004)

[11] Borkent A. World species of biting midges 2. Diptera: Ceratopogonidae (2012).

[12] Mands V Kline DL Blackwell A. *Culicoides* midge trap enhancement with animal odour baits in Scotland. Med Vet Entomol. (2004) 18:36–342. doi: 10.1111/j.0269-283X.2004.00516.x 15641999

[13] Sanders CJ Shortall CR Gubbins S Burgin L Gloster J Harrington R. Influence of season and meteorological parameters on flight activity of *Culicoides* biting midges. J Appl Ecol. (2011) 48:1355–64. doi: 10.1111/j.1365-2664.2011.02051.x

[14] Sarvašová A Kočišová A Halán M Delécolle JC Mathieu B. Morphological and molecular analysis of the genus *Culicoides* (Diptera: Ceratopogonidae) in Slovakia with five new records. Zootaxa. (2014) 3872:541–60. doi: 10.11646/zootaxa.3872.5.6, PMID: 25544100

[15] Slama D Chaker E Mathieu B Babba H Depaquit J Augot D. Biting midges monitoring (Diptera: Ceratopogonidae: *Culicoides* Latreille) in the governate of Monastir (Tunisia): species composition and molecular investigations. Parasitol Res. (2014) 113:2435–43. doi: 10.1007/s00436-014-3873-1, PMID: 24825311

[16] Kremer M Delecolle JP. Variabilité des caractères morphologiques des Culicoides. Ann Parasitol. (1974) 49:617–9. 4478053

[17] Chaker E Kremer M. Les *Culicoides* de Tunisie: particularités morphologiques. Chorologie et écologie des espèces retrouvées. Arch Inst Pasteur Tunis. (1982) 59:511–40. PMID: 7184477

[18] Braverman Y Galun R Ziv M. Breeding sites of some *Culicoides* species (Diptera, Ceratopogonidae) in Israel. Mosq News. (1974) 34:303–8.

[19] Rieb JP Kremer M. Preliminary note on *Culicoides* of Ried of Alsace, with special reference to halophilic species. Mosq News. (1977) 37:288.

[20] Uslu U Dik B. Description of breeding sites of *Culicoides* species (Diptera: Ceratopogonidae) in Turkey. Parasite. (2007) 14:173–7. doi: 10.1051/parasite/2007142173, PMID: 17645192

[21] Zimmer JY Brostaux Y Haubruge E Francis F. Larval development sites of the main *Culicoides* species (Diptera: Ceratopogonidae) in northern Europe and distribution of coprophilic species larvae in Belgian pastures. Vet Parasitol. (2014) 205:676–86. doi: 10.1016/j.vetpar.2014.08.029, PMID: 25241330

[22] Balenghien T Garros C Mathieu B Setier-Rio ML Allène X Gardes L. La surveillance des Culicoïdes en France. Bull Epidemiol Anses. (2010) 35:8–9.

[23] Venail R Balenghien T Guis H Tran A Setier-Rio ML Delécolle JC. Assessing diversity and abundance of vector populations at a national scale: example of *Culicoides* surveillance in France after bluetongue virus emergence In: Mehlhorn H, editor. Parasitol Res Monograph, vol. 3. Berlin, Heidelberg: Springer-Verlag (2012). 77–102.

[24] Ortega MD Holbrook FR Lloyd JE. Seasonal distribution and relationship to temperature and precipitation of the most abundant species of *Culicoides* in five provinces of Andalusia. Spain J Am Mosq Control Assoc. (1999) 15:391–9. PMID: 10480132

[25] Dujardin JP. Morphometrics applied to medical entomology. Infect Genet Evol. (2008) 8:875–90. doi: 10.1016/j.meegid.2008.07.011, PMID: 18832048

[26] Kaba D Berté D Ta BT Tellería J Solano P Dujardin JP. The wing venation patterns to identify single *tsetse* flies. Infect Genet Evol. (2017) 47:132–9. doi: 10.1016/j.meegid.2016.10.008, PMID: 27765637

[27] Dujardin JP Kaba D Solano P Dupraz M McCoy KD Jaramillo-O N. Outline based morphometrics, an overlooked method in arthropod studies? Infect Genet Evol. (2014) 28:704–14. doi: 10.1016/j.meegid.2014.07.035, PMID: 25111609

[28] Dvorak V Aytekin AM Alten B Skarupova S Votypka J Volf P. A comparison of the intraspecific variability of *Phlebotomus sergenti* parrot, 1917 (Diptera: Psychodidae). J Vector Ecol. (2006) 31:229–38. doi: 10.3376/1081-1710(2006)31[229:acotiv]2.0.co;2, PMID: 17249339

[29] Lorenz C Almeida F Almeida-Lopes F Louise C Pereira SN Petersen V. Geometric morphometrics in mosquitoes: what has been measured? Infect Genet Evol. (2017) 54:205–15. doi: 10.1016/j.meegid.2017.06.029, PMID: 28673547

[30] Ebhodaghe F Billah MK Adabie-Gomez D Yahaya A. Morphometric diagnosis of *Glossina palpalis* (Diptera: Glossinidae) population structure in Ghana. BMC Res Notes. (2017) 10:778. doi: 10.1186/s13104-017-3113-827, PMID: 29284545 PMC5746955

[31] Chaiphongpachara T Duvallet G Changbunjong T. Wing phenotypic variation among *Stomoxys calcitrans* (Diptera: Muscidae) populations in Thailand. Insects. (2022) 13:405. doi: 10.3390/insects13050405, PMID: 35621741 PMC9143182

[32] Prudhomme J Cassan C Hide M Toty C Rahola N Vergnes B. Ecology and morphological variations in wings of *Phlebotomus ariasi* (Diptera: Psychodidae) in the region of Roquedur (Gard, France): a geometric morphometrics approach. Parasit Vectors. (2016) 9:578. doi: 10.1186/s13071-016-1872-z, PMID: 27842606 PMC5109773

[33] Prudhomme J Gunay F Rahola N Ouanaimi F Guernaoui S Boumezzough A. Wing size and shape variation of *Phlebotomus papatasi* (Diptera: Psychodidae) populations from the south and north slopes of the Atlas Mountains in Morocco. J Vector Ecol. (2012) 37:137–47. doi: 10.1111/j.1948-7134.2012.00210.x, PMID: 22548547

[34] Kamimura EH Viana MC Lilioso M Fontes FHM Pires-Silva D Valença-Barbosa C. Drivers of molecular and morphometric variation in *Triatoma brasiliensis* (Hemiptera: Triatominae): the resolution of geometric morphometrics for populational structuring on a microgeographical scale. Parasit Vectors. (2020) 13:455. doi: 10.1186/s13071-020-04340-7, PMID: 32894173 PMC7487581

[35] Altunsoy F Ercan I Ocakoglu G. Analysis of morphometric characteristics of different populations of *Tabanus bromius* Linne 1758 (Diptera: Tabanidae). Pak J Zool. (2017) 49:1013–8. doi: 10.17582/journal.pjz/2017.49.3.1013.1018

[36] Chaiphongpachara T Weluwanarak T Changbunjong T. Intraspecific variation in wing geometry among *Tabanus rubidus* (Diptera: Tabanidae) populations in Thailand. Front Vet Sci. (2022) 9:920755. doi: 10.3389/fvets.2022.920755, PMID: 36118331 PMC9480827

[37] Limsopatham K Klong-Klaew T Fufuang N Sanit S Sukontason KL Sukontason K. Wing morphometrics of medically and forensically important muscid flies (Diptera: Muscidae). Acta Trop. (2021) 222:106062. doi: 10.1016/j.actatropica.2021.106062, PMID: 34289390

[38] Muñoz-Muñoz F Talavera S Pagès N. Geometric morphometrics of the wing in the subgenus *Culicoides* (Diptera: Ceratopogonidae): from practical implications to evolutionary interpretations. J Med Entomol. (2011) 48:129–39. doi: 10.1603/me10110, PMID: 21485347

[39] Hajd Henni L Sauvage F Ninio C Depaquit J Augot D. Wing geometry as a tool for discrimination of Obsoletus group (Diptera: Ceratopogonidae: *Culicoides*) in France. Infect Genet Evol. (2014) 21:110–7. doi: 10.1016/j.meegid.2013.10.008, PMID: 24514019

[40] Hadj-Henni L De Meulemeester T Mathieu B Depaquit J Augot D. Taxonomic assessment of *Culicoides brunnicans*, *C. santonicus* and *C. vexans* (Diptera: Ceratopogonidae) in France: implications in systematics. Infect Genet Evol. (2015) 33:324–31. doi: 10.1016/j.meegid.2015.05.024 26005070

[41] Hadj-Henni L Djerada Z Millot C Augot D. Comprehensive characterisation of *Culicoides clastrieri* and *C. festivipennis* (Diptera: Ceratopogonidae) according to morphological and morphometric characters using a multivariate approach and DNA barcode. Sci Rep. (2021) 11:521. doi: 10.1038/s41598-020-78053-3, PMID: 33441647 PMC7806617

[42] Oke PO Samuel OM Oke-Egbodo BE Adejinmi JO Oluwayelu DO. Wing vein shape signal in *Culicoides oxystoma* (Schultzei group) in Nigeria—tool for discrimination (Diptera: Ceratopogonidae) using geometric approach. Zool Anz. (2018) 279:26–37. doi: 10.1016/j.jcz.2018.08.003

[43] Muñoz-Muñoz F Ramoneda J Pagès N Pujol N Talavera S. Is the morphology of *Culicoides* intersexes parasitized by mermithid nematodes a parasite adaptation? A morphometric approach to *Culicoides circumscriptus* (Diptera: Ceratopogonidae). J Invertebr Pathol. (2016) 135:1–9. doi: 10.1016/j.jip.2016.01.008, PMID: 26809123

[44] Muñoz-Muñoz F Pagès N Durao AF England M Werner D Talavera S. Narrow versus broad: sexual dimorphism in the wing form of western European species of the subgenus *Avaritia* (*Culicoides*, Ceratopogonidae). Integ Zool. (2021) 16:769–84. doi: 10.1111/1749-4877.12516, PMID: 33433938

[45] Muñoz-Muñoz F Talavera S Carpenter S Nielsen SA Werner D Pagès N. Phenotypic differentiation and phylogenetic signal of wing shape in western European biting midges, *Culicoides spp*., of the subgenus Avaritia. Med Vet Entomol. (2014) 28:319–29. doi: 10.1111/mve.12042, PMID: 24387691

[46] Jacquemin G. Les marais salés de Lorraine. Premier bilan entomologique (colloque de Besançon 1999). Bull Soci Lorr Entomol. (2001) 8:6–1.

[47] Villard P Muñoz F Balenghien T Balenghein T Baldet T Lancelot R. Modeling *Culicoides* abundance in mainland France: implications for surveillance. Parasit Vectors. (2019) 12:391. doi: 10.1186/s13071-019-3642-1, PMID: 31387649 PMC6683357

[48] Ninio C Augot D Dufour B Depaquit J. Emergence of *Culicoides obsoletus* from indoor and outdoor breeding sites. Vet Parasitol. (2011) 183:125–9. doi: 10.1016/j.vetpar.2011.07.020, PMID: 21840126

[49] Delécolle JC. Nouvelle contribution à l’étude systématique et iconographique des espèces du genre *Culicoides* (Diptera: Ceratopognidae) du Nord-Est de la France. Ph.D. dissertation. Université Louis Pasteur Strasbourg, France (1985).

[50] Augot D Sauvage F Jouet D Simphal E Veuille M Couloux A. Discrimination of *Culicoides obsoletus* and *C. scoticus*, potential bluetongue vectors, by morphometrical and mitochondrial cytochrome oxidase subunit I analysis. Infect Genet Evol. (2010) 10:629–37. doi: 10.1016/j.meegid.2010.03.016, PMID: 20381646

[51] Simon C Frati F Beckenbach A Crespi B Liu H Flook P. Evolution, weighing and phylogenetic unity of mitochondrial gene sequences and a compilation of conserved polymerase chain reaction primers. Ann Entomol Soc Am. (1994) 87:651–701. doi: 10.1093/aesa/87.6.651

[52] Bonfeld JK Staden R. Experiment files and their application during largescale sequencing projects. DNA Seq. (1996) 6:109–17. doi: 10.3109/10425179609010197, PMID: 8907307

[53] Kumar S Stecher G Tamura K. Mega 7: molecular evolutionary genetics analysis Verion 7.0 for bigger datasests. Mol Biol Evol. (2016) 33:1870–4. doi: 10.1093/molbev/msw054, PMID: 27004904 PMC8210823

[54] Augot D Mathieu B Hadj-Henni L Barriel V Zapata Mena S Smolis S. Molecular phylogeny of 42 species of *Culicoides* (Diptera, Ceratopogonidae) from three continents. Parasite. (2017) 24:23. doi: 10.1051/parasite/2017020, PMID: 28643630 PMC5482051

[55] Klingenberg CP. Analyzing fluctuating asymmetry with geometric morphometrics: concepts, methods, and applications. Symmetry. (2015) 7:843–934. doi: 10.3390/sym7020843

[56] Wirth WW Blanton FL. Biting midges of the genus *Culicoides* from Panama (Diptera: Heleidae). Proc United Sates Natl Museum. (1959) 109:237–482. doi: 10.5479/si.00963801.109-3415.237

[57] Dujardin S Dujardin JP. Geometric morphometrics in the cloud. Infect Genet Evol. (2019) 70:189–96. doi: 10.1016/j.meegid.2019.02.018, PMID: 30794886

[58] Bookstein FL. Morphometric tools for landmark data In: Geometry and biology. NY: Cambridge University Press (1991).

[59] Dujardin JP Dujardin S Kaba D Santillán-Guayasamín S Villacís AG Piyaselakul S. The maximum likelihood identification method applied to insect morphometric data. Zool Syst. (2017) 42:46–58. doi: 10.11865/zs.201704

[60] Rohlf FJ. Morphometric spaces, shape components and the effects of linear transformations In: Marcus LF Corti M Loy A GJP N Slice D, editors. Advances in Morphometrics. Plenum publication. New York, NY: NATO ASI, Series A Life Sciences (1996). 117–29.

[61] Changbunjong T Sumruayphol S Weluwanarak T Ruangsittichai J Dujardin JP. Landmark and outline-based geometric morphometrics analysis of three *Stomoxys* flies (Diptera: Muscidae). Folia Parasitol. (2016) 63:2016.037. doi: 10.14411/fp.2016.037, PMID: 27827335

[62] Chaiphongpachara T Laojun S. Effectiveness of landmark-and semi-landmark-based geometric morphometric to identify four species of *Culex* mosquitoes in Thailand. J Adv Vet Anim Res. (2019) 2019:278–83. doi: 10.5455/javar.2019.f345.eCollection PMC6760499 31583223

[63] Gómez GF Márquez EJ Gutiérrez LA Conn JE Correa MM. Geometric morphometric analysis of Colombian *Anopheles albimanus* (Diptera: Culicidae) reveals significant effect of environmental factors on wing traits and presence of a metapopulation. Acta Trop. (2014) 135:75–85. doi: 10.1016/j.actatropica.2014.03.020, PMID: 24704285 PMC4464773

[64] Jirakanjanakit N Leemingsawat S Thongrungkiat S Apiwathnasorn C Singhaniyom S Bellec C. Influence of larval density or food variation on the geometry of the wing of *Aedes (Stegomyia) aegypti*. Tropical Med Int Health. (2007) 12:1354–60. doi: 10.1111/j.1365-3156.2007.01919.x 18045262

[65] Morales-Vargas ER Ya-Umphan P Phumala-Morales N Komalamisra N Dujardin JP. Climate associated size and shape changes in *Aedes aegypti* (Diptera: Culicidae) populations from Thailand. Infect Genet Evol. (2010) 10:580–5. doi: 10.1016/j.meegid.2010.01.004, PMID: 20123039

[66] Ayala D Caro-Riaño H Dujardin JP Rahola N Simard F Fontenille D. Chromosomal and environmental determinants of morphometric variation in natural populations of the malaria vector *Anopheles funestus* in Cameroon. Infect Genet Evol. (2011) 11:940–7. doi: 10.1016/j.meegid.2011.03.003, PMID: 21414420 PMC3665408

[67] Wittmann EJ Baylis M. Climate change: effects on *Culicoides* –transmitted viruses and implications for the UK. Vet J. (2000) 160:107–17. doi: 10.1053/tvjl.2000.0470, PMID: 10985802

[68] Lühken R Steinke S Hoppe N Kiel E. Effects of temperature and photoperiod on the development of overwintering immature *Culicoides chiopterus* and *C. dewulfi*. Vet Parasitol. (2015) 214:195–9. doi: 10.1016/j.vetpar.2015.10.001, PMID: 26467278

[69] Prieto C Dahners HW. Resource utilization and environmental and spatio-temporal overlap of a hilltopping lycaenid butterfly community in the Colombian Andes. J Insect Sci. (2009) 9:16. doi: 10.1673/031.009.1601, PMID: 19613456 PMC3011900

[70] Ray C. The application of Bergmann’s and Allen’s rules to the poikilotherms. J Morphol. (1960) 106:85–108. doi: 10.1002/jmor.1051060104 14436612

[71] Houle D Govindaraju DR Omholt S. Phenomics: the next challenge. Nat Rev Genet. (2010) 11:855–66. doi: 10.1038/nrg2897 21085204

[72] Carvalho LPC Costa GDS Pereira Júnior AM de Paulo PFM Silva GS Carioca ALPM. DNA barcoding of genus *Culicoides* biting midges (Diptera: Ceratopogonidae) in the Brazilian Amazon. Acta Trop. (2022) 235:106619. doi: 10.1016/j.actatropica.2022.106619, PMID: 35905777

[73] Hajibabaei M Singer GA Hebert PD Hickey DA. DNA barcoding: how it complements taxonomy, molecular phylogenetics and population genetics. Trends Genet. (2007) 23:167–72. doi: 10.1016/j.tig.2007.02.001, PMID: 17316886

[74] Martínez-de la Puente J Martínez J Ferraguti M Morales-de la Nuez A Castro N Figuerola J. Genetic characterization and molecular identification of the bloodmeal sources of the potential bluetongue vector *Culicoides obsoletus* in the Canary Islands, Spain. Parasit Vectors. (2012) 5:147. doi: 10.1186/1756-3305-5-147, PMID: 22827913 PMC3425321

[75] Aguilar-Vega C Rivera B Lucientes J Gutiérrez-Boada I Sánchez-Vizcaíno JM. A study of the composition of the Obsoletus complex and genetic diversity of *Culicoides obsoletus* populations in Spain. Parasit Vectors. (2021) 14:351. doi: 10.1186/s13071-021-04841-z, PMID: 34217330 PMC8254917

[76] Fujita MK Leache AD Burbrink FT McGuire JA Moritz C. Coalescent-based species delimitation in an integrative taxonomy. Trends Ecol Evol. (2012) 27:480–8. doi: 10.1016/j.tree.2012.04.012, PMID: 22633974

[77] Gopurenko D Bellis GA Yanase T Wardhana AH Thepparat A Wang JL. Using integrative taxonomy to investigate species boundaries within *Culicoides* (Diptera: Ceratopogonidae): a case study using subgenus *Avaritia* from Australasia and eastern Asia. Vet Ital. (2015) 51:345–37. doi: 10.1186/s13071-020-04568-3 26741249

[78] Pilgrim J Siozios S Baylis M Venter G Garros C Hurst GDD. *Cardinium* symbiosis as a potential confounder of mtDNA based phylogeographic inference in *Culicoides imicola* (Diptera: Ceratopogonidae), a vector of veterinary viruses. Parasit Vectors. (2021) 14:100. doi: 10.1186/s13071-020-04568-3, PMID: 33557932 PMC7869521

[79] Gopurenko D Bellis G Pengsakul T Siriyasatien P Thepparat A. DNA barcoding of *Culicoides* Latreille (Diptera: Ceratopogonidae) from Thailand reveals taxonomic inconsistencies and novel diversity among reported sequences. J Med Entomol. (2022) 59:1960–70. doi: 10.1093/jme/tjac142, PMID: 36189978

[80] Shults P Hopken M Eyer PA Blumenfeld A Mateos M Cohnstaedt LW. Species delimitation and mitonuclear discordance within a species complex of biting midges. Sci Rep. (2022) 12:1730. doi: 10.1038/s41598-022-05856-x, PMID: 35110675 PMC8810881

[81] Chaker E Delécolle JC Kremer M. Variabilité des caractères morphologiques de *Culicoides circumscriptus* Kieffer 1918. Mise en synonymie de *C. kirovabadicus* Dzhafarov 1964. Arch Inst Pasteur Tunis. (1980) 57:33–40. PMID: 7469615

[82] Phanitchat T Apiwathnasorn C Sungvornyothin S Samung Y Dujardin S Dujardin JP. Geometric morphometric analysis of the effect of temperature on wing size and shape in *Aedes albopictus*. Med Vet Entomol. (2019) 33:476–84. doi: 10.1111/mve.12385, PMID: 31125148

[83] Champakaew D Junkum A Sontigun N Sanit S Limsopatham K Saeung A. Geometric morphometric wing analysis as a tool to discriminate female mosquitoes from different suburban areas of Chiang Mai province, Thailand. PLoS One. (2021) 16:e0260333. doi: 10.1371/journal.pone.0260333, PMID: 34843516 PMC8629303

[84] Abubekerov LA Mullens BA. Egg and larval morphology of *Culicoides sonorensis* (Diptera: Ceratopogonidae). J Med Entomol. (2018) 55:553–60. doi: 10.1093/jme/tjx236, PMID: 29281109

[85] Erram D Burkett-Cadena N. Oviposition of *Culicoides insignis* (Diptera: Ceratopogonidae) under laboratory conditions with notes on the developmental life history traits of its immature stages. Parasit Vectors. (2021) 14:522. doi: 10.1186/s13071-021-05025-5, PMID: 34627349 PMC8501582

