# Supplementary material for: Wing morphology variations in Culicoides circumscriptus from France
Source: Front Vet Sci. 2023 Apr 24;10:1089772. doi: 10.3389/fvets.2023.1089772 (PMC10164937; doi:10.3389/fvets.2023.1089772)
Supplement: Supplementary file 1 [file Table_1.docx]

Supplemental data Table S1. Estimation of pairwise distance (±SD) of *Culicoides circumscriptus* populations for the cytochrome c oxidase subunit I (COI) domain of mitochondrial DNA. We included the following GenBank sequences: Algeria (KX853263-KX853269), China (MK917493-494), Greece (MZ695768), India (KF145180, OM060693-95), Israel (MK331727), Lithuania (MW207304), Morocco (MK732290), Portugal (AF069248), Slovakia (KJ624071, OM665424), Spain (HM241850-HM241855), Sweden (JQ620049-JQ620054, JQ978443), Switzerland (HQ824460-HQ824465), Tunisia (KJ729969-KJ729972) and Turkey (MF105752-MF105756, MF594384-MF594386), *Culicoides* *nubeculosus* (KJ624102) was used as an out group. The specimens from this study are shown in bold.

|  |  | **1** | **2** | **3** | **4** | **5** | **6** | **7** | **8** | **9** | **10** | **11** | **12** | **13** | **14** | **15** | **16** | **17** | **18** |
| --- | --- | --- | --- | --- | --- | --- | --- | --- | --- | --- | --- | --- | --- | --- | --- | --- | --- | --- | --- |
| *C. circumscriptus* | **1**-Algeria |  | 0.022 | 0.004 | 0.020 | 0.006 | 0.008 | 0.004 | 0.026 | 0.006 | 0.003 | 0.004 | 0.022 | 0.005 | 0.006 | **0.006** | **0.007** | **0.007** | 0.025 |
|  | **2**-China | 0.172 |  | 0.022 | 0.020 | 0.022 | 0.022 | 0.022 | 0.024 | 0.021 | 0.022 | 0.021 | 0.022 | 0.023 | 0.021 | **0.021** | **0.022** | **0.021** | 0.022 |
|  | **3**-Greece | 0.010 | 0.166 |  | 0.019 | 0.005 | 0.007 | 0.003 | 0.026 | 0.005 | 0.003 | 0.003 | 0.022 | 0.004 | 0.005 | **0.005** | **0.006** | **0.006** | 0.025 |
|  | **4**-India | 0.143 | 0.156 | 0.136 |  | 0.020 | 0.020 | 0.019 | 0.025 | 0.019 | 0.019 | 0.019 | 0.019 | 0.019 | 0.018 | **0.019** | **0.018** | **0.019** | 0.025 |
|  | **5**-Israel | 0.020 | 0.169 | 0.010 | 0.145 |  | 0.009 | 0.006 | 0.026 | 0.007 | 0.006 | 0.006 | 0.022 | 0.006 | 0.007 | **0.006** | **0.008** | **0.007** | 0.025 |
|  | **6**-Lithuania | 0.030 | 0.169 | 0.020 | 0.142 | 0.031 |  | 0.008 | 0.026 | 0.007 | 0.007 | 0.006 | 0.021 | 0.008 | 0.007 | **0.006** | **0.008** | **0.007** | 0.026 |
|  | **7**-Morocco | 0.010 | 0.169 | 0.005 | 0.138 | 0.015 | 0.025 |  | 0.025 | 0.006 | 0.003 | 0.004 | 0.023 | 0.005 | 0.006 | **0.006** | **0.007** | **0.007** | 0.025 |
|  | **8**-Portugal | 0.205 | 0.193 | 0.203 | 0.210 | 0.209 | 0.209 | 0.199 |  | 0.025 | 0.025 | 0.025 | 0.026 | 0.026 | 0.025 | **0.025** | **0.025** | **0.025** | 0.010 |
|  | **9**-Slovakia | 0.024 | 0.161 | 0.018 | 0.134 | 0.026 | 0.028 | 0.020 | 0.203 |  | 0.005 | 0.005 | 0.021 | 0.006 | 0.005 | **0.006** | **0.007** | **0.006** | 0.025 |
|  | **10**-Spain | 0.010 | 0.167 | 0.005 | 0.136 | 0.015 | 0.023 | 0.005 | 0.200 | 0.019 |  | 0.004 | 0.022 | 0.004 | 0.005 | **0.005** | **0.007** | **0.006** | 0.025 |
|  | **11**-Sweden | 0.018 | 0.163 | 0.012 | 0.137 | 0.022 | 0.025 | 0.015 | 0.201 | 0.022 | 0.014 |  | 0.021 | 0.005 | 0.004 | **0.005** | **0.006** | **0.006** | 0.025 |
|  | **12**-Switzerland | 0.175 | 0.173 | 0.171 | 0.135 | 0.178 | 0.162 | 0.177 | 0.223 | 0.172 | 0.175 | 0.170 |  | 0.023 | 0.021 | **0.021** | **0.022** | **0.021** | 0.026 |
|  | **13**-Tunisia | 0.025 | 0.182 | 0.018 | 0.150 | 0.024 | 0.037 | 0.020 | 0.218 | 0.032 | 0.019 | 0.027 | 0.192 |  | 0.006 | **0.006** | **0.007** | **0.007** | 0.025 |
|  | **14**-Turkey | 0.027 | 0.159 | 0.020 | 0.134 | 0.030 | 0.029 | 0.025 | 0.204 | 0.022 | 0.024 | 0.022 | 0.170 | 0.035 |  | **0.005** | **0.006** | **0.005** | 0.025 |
|  | **15-France (Corsica)** | **0.026** | **0.157** | **0.018** | **0.135** | **0.026** | **0.027** | **0.022** | **0.198** | **0.026** | **0.021** | **0.023** | **0.171** | **0.033** | **0.023** |  | **0.006** | **0.004** | **0.024** |
|  | **16-France (Moselle)** | **0.027** | **0.163** | **0.018** | **0.126** | **0.028** | **0.031** | **0.023** | **0.196** | **0.027** | **0.021** | **0.022** | **0.171** | **0.033** | **0.025** | **0.025** |  | **0.007** | **0.025** |
|  | **17-France (Var)** | **0.030** | **0.154** | **0.021** | **0.136** | **0.027** | **0.028** | **0.025** | **0.201** | **0.025** | **0.024** | **0.025** | **0.172** | **0.036** | **0.021** | **0.017** | **0.026** |  | **0.025** |
| 18-*C. nubeculosus* (out group) | | 0.195 | 0.177 | 0.193 | 0.203 | 0.199 | 0.199 | 0.189 | 0.041 | 0.194 | 0.190 | 0.193 | 0.224 | 0.208 | 0.196 | **0.191** | **0.193** | **0.194** |  |
