# Supplementary material for: Wing morphology variations in Culicoides circumscriptus from France
Source: Front Vet Sci. 2023 Apr 24;10:1089772. doi: 10.3389/fvets.2023.1089772 (PMC10164937; doi:10.3389/fvets.2023.1089772)
Supplement: Supplementary file 2 [file Presentation_1.pptx]

## Slide 1
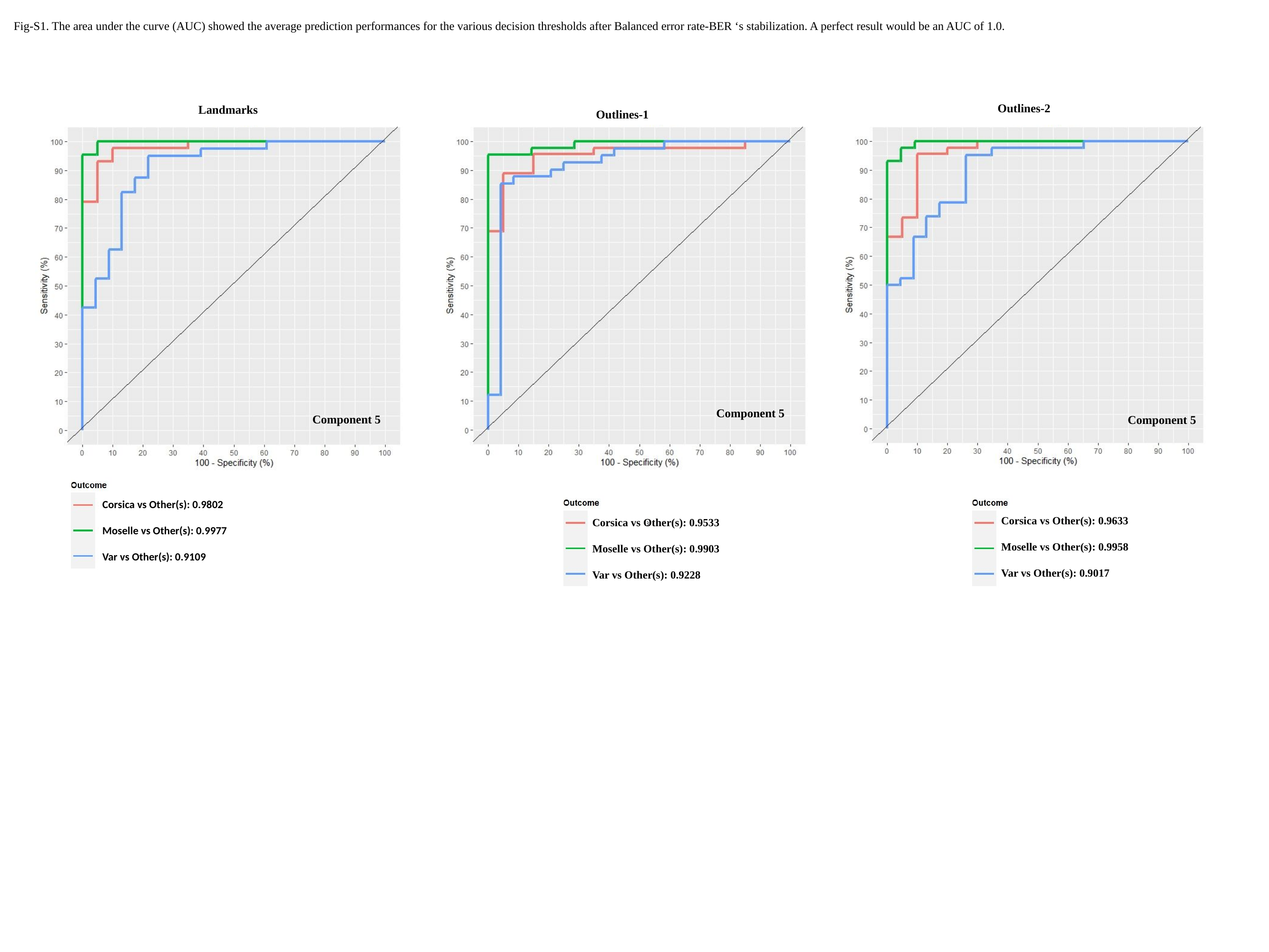

Fig-S1. The area under the curve (AUC) showed the average prediction performances for the various decision thresholds after Balanced error rate-BER ‘s stabilization. A perfect result would be an AUC of 1.0.
Outlines-2
Component 5
Corsica vs Other(s): 0.9633
Moselle vs Other(s): 0.9958
Var vs Other(s): 0.9017
Landmarks
Component 5
Corsica vs Other(s): 0.9802
Moselle vs Other(s): 0.9977
Var vs Other(s): 0.9109
Outlines-1
Component 5
Corsica vs Other(s): 0.9533
Moselle vs Other(s): 0.9903
Var vs Other(s): 0.9228
